# Biomarkers for Predicting Malignant Transformation of Premalignant Lesions of the Larynx: A Systematic Review †

**DOI:** 10.3390/diagnostics16020236

**Published:** 2026-01-12

**Authors:** Juan P. Rodrigo, Reydson Alcides de Lima-Souza, Fernando López, Göran Stenman, Abbas Agaymy, Miquel Quer, Vinidh Paleri, Ilmo Leivo, Alfons Nadal, Nina Zidar, Fernanda V. Mariano, Henrik Hellquist, Nina Gale, Alfio Ferlito

**Affiliations:** 1 Department of Otolaryngology, Hospital Universitario Central de Asturias, University of Oviedo, Instituto de Investigación Sanitaria del Principado de Asturias, Instituto Universitario de Oncología del Principado de Asturias, 33011 Oviedo, Spain; lopezfernando@uniovi.es; 2 CIBER de Cáncer (CIBERONC), 28029 Madrid, Spain; 3 Department of Oral Diagnosis, Faculdade de Odontologia de Piracicaba, Universidade Estadual de Campinas (UNICAMP), Piracicaba 13414-903, SP, Brazil; reydsonalsouza@gmail.com; 4 Department of Pathology, Sahlgrenska Center for Cancer Research, Sahlgrenska University Hospital, University of Gothenburg, 405 30 Gothenburg, Sweden; goran.stenman@llcr.med.gu.se; 5 Institute of Pathology, University Hospital Erlangen, 91054 Erlangen, Germany; al_ijaimy@yahoo.com; 6 Department of Otolaryngology, Hospital Santa Creu y Sant Pau, 08041 Barcelona, Spain; mquer@santpau.cat; 7 The Royal Marsden NHS Foundation Trust, London SW3 6JJ, UK; vinidh.paleri@rmh.nhs.uk; 8 Institute of Biomedicine, Pathology, University of Turku, Turku University Hospital, 20520 Turku, Finland; ilmo.leivo@utu.fi; 9 Pathology Department, Clínic Barcelona, Basic Clinical Practice Department, Universitat de Barcelona, Institut d’Investigacions Biomèdiques August Pi i Sunyer, 08007 Barcelona, Spain; anadal@clinic.cat; 10 Institute of Pathology, Faculty of Medicine, University of Ljubljana, Korytkova 2, 1000 Ljubljana, Slovenia; nina.zidar@mf.uni-lj.si (N.Z.); nina.gale@mf.uni-lj.si (N.G.); 11 Department of Pathology, School of Medical Sciences, University of Campinas (UNICAMP), Campinas 13083-970, SP, Brazil; fevimariano@gmail.com; 12 Department of Biomedical Sciences and Medicine, Algarve Biomedical Center Research Institute, University of Algarve, 8005-139 Faro, Portugal; henrikhellquist@pm.me; 13 Department of Cellular Pathology, Northern Lincolnshire and Goole NHS Foundation Trust, Lincoln LN2 5QY, UK; 14 International Head and Neck Scientific Group, 35100 Padua, Italy; profalfioferlito@gmail.com

**Keywords:** laryngeal dysplasia, premalignant lesions, biomarkers, malignant transformation, systematic review, ROBINS-I

## Abstract

**Background/Objectives**: Premalignant laryngeal lesions carry a variable risk of malignant transformation to squamous cell carcinoma. Identifying reliable biomarkers that predict malignant transformation could improve patient management and surveillance strategies. The objective of this work is to perform a systematic review of the literature on biomarkers that predict malignant transformation of premalignant laryngeal lesions. **Methods**: We conducted a systematic review following PRISMA 2020 guidelines. The PubMed, Scopus and Embase databases, and Google Scholar were searched for studies published between January 2011 and November 2025. Studies investigating biomarkers that predict malignant transformation of histopathologically confirmed premalignant laryngeal lesions were included. Risk of bias was assessed using the ROBINS-I tool. **Results**: From 166 initially identified records, 11 studies met the inclusion criteria, including 730 patients. These studies investigated diverse biomarker categories such as protein markers (cortactin, FAK, NANOG, SOX2, CSPG4), immune markers (tumor-infiltrating lymphocytes, immune gene signatures), microRNAs (miR-183-5p, miR-155-5p, miR-106b-3p), and genetic markers (chromosomal instability, *PIK3CA* amplification and mutations, *FGFR3* mutations). Five studies provided adequate follow-up data on transformation outcomes. Most studies showed a moderate to serious risk of bias primarily due to limited confounder control and incomplete reporting. **Conclusions**: While several promising biomarker candidates have been identified, the evidence base remains limited due to small sample sizes, heterogeneous methodologies, and inadequate follow-up data. Cortactin/FAK protein expression and immune signatures are the most promising but require validation in larger, well-designed prospective cohorts.

## 1. Introduction

Laryngeal cancer represents a significant global health burden, with squamous cell carcinoma (SCC) accounting for most cases [1]. The development of laryngeal cancer typically follows a multistep progression from normal epithelium through premalignant lesions to invasive carcinoma. Premalignant laryngeal lesions, with different clinical presentations such as leukoplakia and erythroplakia, present a clinical challenge due to their variable and unpredictable potential for malignant transformation and inconsistency of diagnostic criteria across different studies [2,3]. In the current World Health Organization (WHO) classification, laryngeal premalignant lesions are conceptualized as squamous intraepithelial lesions showing architectural and cytological atypia without stromal invasion. Recent WHO editions and the European Laryngological Society (ELS) endorse a predominantly two-tier system, grouping lesions into low-grade and high-grade dysplasia, with carcinoma in situ reserved as an optional third category for lesions with full-thickness atypia. This framework aims to improve diagnostic reproducibility while maintaining a meaningful correlation with malignant transformation risk [2,3].

Reported rates of malignant transformation of laryngeal premalignant lesions vary considerably, ranging from 5% to 30%, depending on the degree of dysplasia, anatomical location, and patient risk factors [4]. This wide risk variation creates uncertainty in the clinical management, with some patients requiring intensive surveillance, whereas others may be managed more conservatively (i.e., less intensive surveillance) [5].

From a diagnostic standpoint, evaluation of suspected premalignant laryngeal lesions relies on high-definition white-light laryngoscopy, often complemented by videostroboscopy and enhanced endoscopic modalities such as narrow-band imaging (NBI) or contact endoscopy. Once a biopsy is obtained, current clinical practice depends on histopathological assessment of dysplasia grade to stratify risk, but this approach has significant limitations [2,5]. Histopathological grading shows considerable interobserver variability, and the correlation between dysplasia grade and transformation risk is far from perfect [4]. Moreover, some premalignant lesions with low-grade dysplasia can progress to malignancy, while others with high-grade dysplasia may remain stable or even regress [5].

The identification of reliable molecular biomarkers that predict the risk and dynamics of malignant transformation in premalignant laryngeal lesions would significantly improve clinical management. Such biomarkers could guide decisions on surveillance intervals, treatment strategies, and patient counselling. Research has explored diverse biomarker categories, including protein expression, genetic alterations, immune signatures, and epigenetic modifications [4,5]. Existing reviews are either outdated or limited in scope, underscoring the need for a comprehensive synthesis of current evidence [6].

Therefore, this review aims to determine whether, in adults with histologically confirmed premalignant laryngeal lesions, molecular, genetic, epigenetic, protein-expression, immune, or imaging-based biomarkers can reliably predict progression to invasive SCC compared with their absence or reliance solely on histopathological grading. The primary objective is to identify and critically evaluate biomarkers reported in the past 15 years that predict malignant transformation. Secondary objectives include assessing methodological quality, highlighting the most promising biomarker candidates, identifying gaps in the literature, and proposing recommendations for future research.

## 2. Materials and Methods

This systematic review was conducted and reported in accordance with the Preferred Reporting Items for Systematic Reviews and Meta-Analyses (PRISMA) 2020 statement [7]. The review protocol was developed a priori and is registered in Open Science Framework (https://osf.io/7txzj (accessed on 20 November 2025)).

### 2.1. Eligibility Criteria

The study PECOS (i.e., population, exposure, comparison, outcomes, studies) framework was developed to guide the focused review question and to establish the inclusion criteria as follows: P (Population): patients of any age with histopathologically confirmed premalignant laryngeal lesions, including mild, moderate, or severe dysplasia and carcinoma in situ; E (Exposure): assessment of biomarkers (protein, genetic, epigenetic, immune, or microRNA markers) evaluated for their potential to predict malignant transformation; C (Comparison): patients with premalignant lesions that did not progress to invasive carcinoma, or comparison across biomarker expression levels or molecular profiles; O (Outcomes): malignant transformation outcomes, including time to transformation, recurrence, and diagnostic performance metrics (e.g., sensitivity, specificity, predictive values, hazard ratios, odds ratios); S (Study types): observational human studies (prospective or retrospective cohort, case–control, or cross-sectional) published in peer-reviewed indexed journals, between January 2011 and November 2025, with available follow-up data and sufficient methodological description for data extraction.

The exclusion criteria were as follows: (1) studies involving animal models, in vitro experiments, or cell lines; (2) articles evaluating benign lesions without dysplasia or studies addressing invasive laryngeal cancer without analysis of premalignant components; (3) studies lacking follow-up data on malignant transformation or without extractable biomarker outcomes; (4) case reports, case series with fewer than 10 patients, reviews, editorials, letters, conference abstracts, short communications, book chapters, or protocol papers; (5) studies not published in English; (6) full-text not available; and (7) duplicated cohorts or overlapping samples.

### 2.2. Information Sources and Search Strategy

We conducted comprehensive searches across three bibliographic databases (PubMed, Scopus, Embase) and one search engine (Google Scholar) to identify relevant studies (Supplementary Table S1). In PubMed, two complementary search strategies were employed. In Google Scholar also two search strategies were used with different keyword combinations. In Scopus and Embase only one search strategy was used. The search was limited to studies published between 1 January 2011, and 23 November 2025, to focus on contemporary biomarker research. Last search was performed on 23 November 2025. No language restrictions were applied during database searches; however, only English-language articles were eligible for inclusion.

### 2.3. Study Selection Process

**Figure 1:**
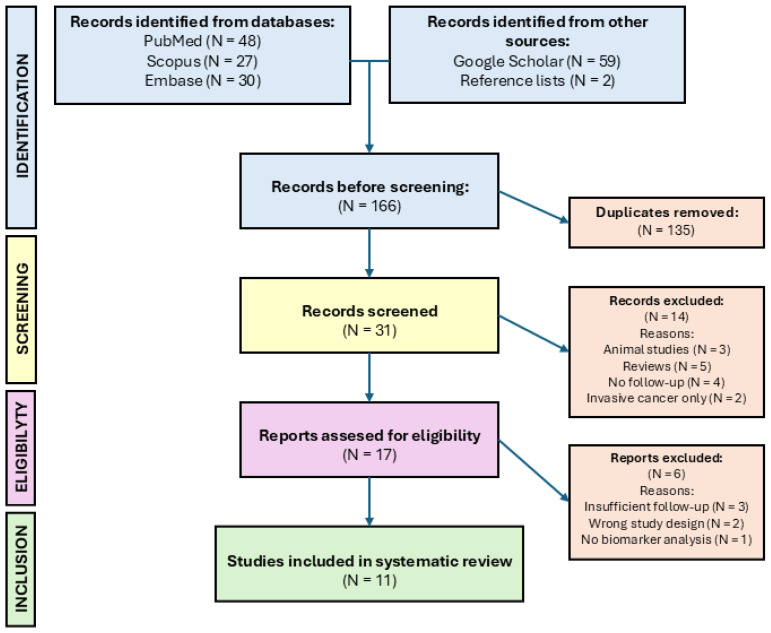
PRISMA flow diagram for study selection process.

One researcher (JPR) reviewed the search results to identify potentially eligible studies. The study selection process was carried out in two stages (Figure 1). In the first stage, titles and abstracts of all retrieved records were screened against the predefined inclusion and exclusion criteria using a structured approach and standardized decision rules. In the second stage, studies that met the initial criteria underwent a full-text review to confirm eligibility. Full texts were obtained whenever possible, and studies were excluded if the complete text could not be accessed. All reasons for exclusion at each stage were systematically documented to ensure transparency and facilitate reporting in the PRISMA flow diagram (Figure 1).

### 2.4. Data Collection and Data Items

Data extraction was performed systematically using a standardized form that captured the following information:

#### 2.4.1. Study Characteristics

First author, year of publication, journal, study design (cohort, case–control, cross-sectional), country or setting, sample size and participant characteristics, and follow-up duration.

#### 2.4.2. Population Characteristics

Patient demographics (age, sex distribution), type and grade of premalignant lesions, anatomical location within the larynx, and relevant risk factors (smoking, alcohol consumption, human papillomavirus—HPV-status).

#### 2.4.3. Biomarker Details

Specific biomarkers investigated, detection methods (immunohistochemistry, PCR, sequencing), tissue type and collection procedures, and cut-off values or scoring criteria.

### 2.5. Outcome Measures

Definition of malignant transformation, number and percentage of patients who experienced transformation, time to transformation, diagnostic accuracy metrics (sensitivity, specificity, positive predictive value, negative predictive value), and effect estimates (hazard ratios, odds ratios) with confidence intervals.

### 2.6. Risk of Bias Assessment

The methodological quality and risk of bias of included studies were evaluated by the first author (JPR) using the Risk of Bias in Non-randomized Studies of Interventions (ROBINS-I) tool [8]. This instrument assesses seven domains of bias: confounding, selection of participants, classification of interventions, deviations from intended interventions, missing data, measurement of outcomes, and selection of reported results. Each domain was rated as low, moderate, serious, or critical risk of bias, or as having insufficient information. An overall judgment of risk of bias was assigned to each study based on the most critical domain. ROBINS-I was selected because most included studies were observational cohorts evaluating prognostic biomarker performance.

### 2.7. Data Synthesis

Given the heterogeneity in biomarkers, study designs, and outcome measures, a qualitative synthesis approach was adopted. Studies were grouped by biomarker type, and findings were summarized descriptively. Where possible, quantitative data on diagnostic accuracy were extracted and presented in summary tables. Although meta-analysis was considered for studies with comparable biomarkers and adequate follow-up data, it was ultimately not performed due to insufficient homogeneity in study populations, biomarker definitions, and outcome measures.

## 3. Results

### 3.1. Study Selection

The database searches yielded a total of 166 studies. After removing duplicates, 31 were screened by title and abstract against the eligibility criteria. Of these, 17 studies were considered potentially eligible and sought full-text retrieval. Following the full-text review, six studies were excluded for reasons such as insufficient follow-up data, inappropriate study design, or absence of biomarker analysis (Supplementary Table S2). Ultimately, 11 studies met the inclusion criteria for this systematic review [9,10,11,12,13,14,15,16,17,18,19]. The study selection process is summarized in the PRISMA flow diagram (Figure 1).

### 3.2. Study Characteristics

The 11 included studies comprised a total of 730 patients with premalignant laryngeal lesions. These studies were published between 2011 and 2025, with the majority (8 studies) appearing during the last seven years, reflecting the growing interest in biomarker research for laryngeal premalignancy.

#### 3.2.1. Study Designs and Settings

The included studies employed diverse designs: one prospective cohort study [19], six retrospective cohort studies [9,10,11,12,14,18], three case–control studies [13,15,16], and one cross-sectional study [17]. The research was conducted across multiple countries, including Spain, Italy, and the Netherlands in Europe, Turkey and China in Asia, as well as in other regions. Sample sizes ranged from 30 to 109 patients, with most investigations being relatively small, single-center studies.

#### 3.2.2. Population Characteristics

Participants were patients with histologically confirmed premalignant laryngeal lesions, such as mild, moderate or severe dysplasia, and carcinoma in situ. Demographic data were incompletely reported in several studies. However, available information indicated predominantly middle-aged to elderly populations with a male predominance, consistent with the epidemiology of laryngeal pathology (Table 1).

#### 3.2.3. Biomarker Categories and Findings

The studies explored multiple biomarker classes (Table 1):

- Protein expression markers:

Cortactin (CTTN) and Focal Adhesion Kinase (FAK) were evaluated by Rodrigo et al. [9], demonstrating that FAK overexpression independently predicted cancer development (HR = 3.706, 95% CI: 1.735–7.916, *p* = 0.001), with combined FAK and CTTN expression showing even stronger predictive value (HR = 5.042, 95% CI: 2.255–11.274, *p* < 0.001). The same group later validated these results in a larger and different cohort of patients, confirming the predictive value of FAK and CTTN expression in malignant transformation of laryngeal dysplasia [12]. NANOG expression, assessed by Rodrigo et al. [11], was associated with an increased risk of transformation (HR = 1.826; 95%CI, 1.222–2.728; *p* = 0.003), and was validated across two independent cohorts. Granda-Díaz et al. [14], in a study of 94 laryngeal dysplasia specimens, demonstrated that SOX2 expression was an independent predictor of laryngeal cancer development (HR = 3.531, 95% CI 1.144 to 10.904; *p* = 0.028). In a study by Shi et al. [19], CSPG4 overexpression correlated with higher recurrence and risk of tumorigenesis following surgical intervention.

- Genetic and chromosomal biomarkers:

Bergshoeff et al. [10] examined chromosome instability and cell cycle proteins (TP53, Cyclin D1); Patients with lesions showing chromosome instability had significantly worse 5-year progression-free survival than those with premalignancies without chromosome instability (*p* = 0.002). Neither histopathology nor the protein markers predicted progression in univariate analysis. Manterola et al. [13] identified a mutational profile in laryngeal dysplasias associated with the risk of progression: mutations in *PIK3CA* and *FGFR3* were detected in cases that progressed to carcinoma but were absent in non-progressing cases. In contrast, mutations in *JAK3*, *MET* and *FBXW7* were found only in non-progressing cases. Similarly, amplification of *PIK3CA* was found to be associated with progression to laryngeal cancer in a study by Montoro-Jiménez et al. (HR = 2.64, 95% CI 1.09–6.37, *p* = 0.031) [18]. In this study, *SOX2* amplification was also associated with increased risk of malignant progression; combining *PIK3CA* and *SOX2* amplification allowed for distinction of three cancer risk subgroups, and *PIK3CA* and *SOX2* co-amplification was the strongest predictor (HR = 2.64, 95% CI 1.09–6.37, *p* = 0.031).

- Immune and inflammatory markers:

Chu et al. [16] showed that high infiltration of lymphocytes (TILs) is significantly correlated with a higher risk of malignant transformation and identified non-brisk TIL patterns as protective (OR = 0.16, 95% CI: 0.03–0.5, *p* = 0.008). And Maffini et al. [17], also identified immune gene signatures (*CCR6*, *CD83*, *HLA-DPB1*, *MX1*, *SNAI1*, *RORC*) associated with risk of progression to cancer.

- MicroRNA signatures:

Tuncturk et al. [15] identified differentially expressed miRNAs in premalignant lesions (miR-183-5p, miR-155-5p, miR-106b-3p, miR-21-5p, miR-218-3p, miR-210-3p) as potential biomarkers for an increased risk of malignant transformation.

### 3.3. Risk of Bias Assessment

**Figure 2:**
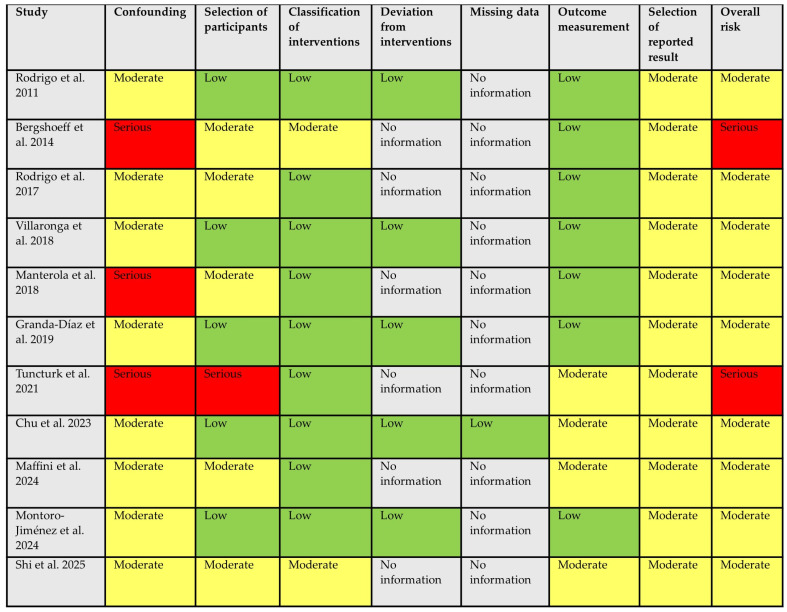
ROBINS-I Risk of bias assessment of the included studies [9,10,11,12,13,14,15,16,17,18,19].

ROBINS-I evaluation revealed methodological limitations across studies (Figure 2). Overall, nine studies (81.2%) had a moderate risk of bias, and two (18.8%) had serious risk; none were rated as critical. Confounding was the most problematic domain, with inadequate adjustment for smoking, alcohol use, HPV status, and lesion grade, while participant selection and outcome measurement were generally adequate. A critical methodological gap was identified across the included studies: 10 of 11 studies (90.9%) failed to report on missing data, including the extent of missing biomarker measurements, loss to follow-up, or methods for handling missing values. This lack of transparency represents a significant threat to internal validity, as missing data may be non-random and related to both biomarker expression and transformation outcomes. The potential for attrition bias and selection bias cannot be adequately assessed without this information.

### 3.4. Evidence Synthesis

Across studies, the most consistently associated biomarkers were cortactin/FAK protein expression, immune gene signatures, SOX2 expression, and NANOG expression, based on effect estimates and methodological rigor. However, limitations such as small sample sizes, short or incomplete follow-up, heterogeneous methodologies, lack of independent validation (except in the case of FAK/cortactin expression), and incomplete control of confounders were common.

### 3.5. Quantitative Synthesis

Meta-analysis was not feasible due to substantial heterogeneity in biomarkers, study populations, and outcome measures. Only five studies [9,11,12,14,18] provided sufficient follow-up data, but differences in biomarker definitions and methodologies precluded meaningful pooling.

## 4. Discussion

This systematic review identified 11 studies evaluating biomarkers for predicting malignant transformation of premalignant laryngeal lesions. The evidence encompasses diverse biomarker categories, including protein expression markers [9,11,12,14,19], genetic markers [10,13,18], immune signatures [16,17], and microRNAs [15]. While several promising candidates were identified, the overall quality of evidence remains limited due to small sample sizes, methodological heterogeneity, and lack of independent validation. The strongest evidence supports cortactin and FAK protein expression as predictive biomarkers, based on a well-designed cohort study with multivariable analysis and robust effect estimates [9]. Moreover, these markers were the only validated in an independent multicenter patient cohort [12]. SOX2 [14] and NANOG [11] expression also show promising value as predictive biomarkers, but they must be validated in multi-center studies. Immune signatures, particularly TIL patterns and immune gene expression profiles, represent an emerging and promising area of investigation [16,17].

Since histopathological grading shows considerable inter-observer variability [20], and the correlation between dysplasia grade and transformation risk is far from perfect [21,22], reliable biomarkers for predicting malignant transformation could significantly influence clinical practice in three key areas: (1) improved risk stratification by complementing histopathology, enabling earlier identification of high-risk patients; (2) surveillance optimization through individualized follow-up intervals; and (3) informed treatment decision-making, potentially reducing overtreatment. Notably, despite increasing biomarker research, no molecular, immunologic, or genomic biomarker has been incorporated into major clinical guidelines, including National Comprehensive Cancer Network (NCCN), ELS, or WHO classifications for laryngeal dysplasia. Current recommendations continue to rely exclusively on histopathological grading and clinical judgment, reflecting insufficient validation, lack of standardized assays, and limited external reproducibility. This gap reinforces the urgent need for clinically actionable biomarkers supported by high-quality prospective evidence.

Collectively, the identified biomarkers may offer mechanistic insight into early laryngeal carcinogenesis.

The predictive value of cortactin and FAK [9,12] suggests that alterations in cell adhesion and motility are early events in laryngeal carcinogenesis, and the association of malignant transformation with NANOG [11] and SOX2 [14] expression supports the cancer stem cell hypothesis, indicating that acquisition of stem cell-like properties may increase the transformation potential. The role of TILs and immune gene signatures [16,17] highlights the importance of immune surveillance in cancer prevention and the potential for immunotherapeutic strategies. Moreover, differential expression of microRNAs between benign, premalignant, and malignant lesions suggests epigenetic dysregulation as a possible driver of transformation and a potential target [15].

Earlier reviews in this field were limited in scope or outdated [6,23]. This review provides an updated and comprehensive synthesis of biomarker evidence for laryngeal premalignant lesions. Compared to previous work, this review reveals expansion in biomarker types, with increased focus on immune markers and multi-gene signatures [15,16,17], and improved methodological approaches in recent studies, including validation cohorts [12] and genomic profiling [13]. However, persistent challenges in study design and reporting hinder clinical translation.

Several limitations of this study should be acknowledged. A major limitation across the reviewed studies is the inadequate adjustment for established risk factors that may confound biomarker associations with malignant transformation. Specifically, most studies did not adequately control for smoking exposure (duration, intensity, cessation status), alcohol consumption patterns, or HPV status—all of which independently influence transformation risk. This confounding may lead to overestimation or underestimation of biomarker predictive value. Future studies must incorporate comprehensive adjustment for these factors through multivariable modeling or stratified analyses to isolate the independent predictive capacity of candidate biomarkers. Another limitation is the definition of “malignant transformation” heterogeneity: The included studies employed varying definitions of malignant transformation, including differences in minimum follow-up duration required to classify a lesion as non-progressive, whether carcinoma in situ is classified as transformation or a premalignant state, and criteria for histological confirmation of progression. This heterogeneity complicates cross-study comparisons and meta-analysis. Despite comprehensive database searches, relevant studies may have been missed, particularly non-English publications or those in regional journals. Studies with negative or inconclusive results may be underrepresented; the small number of included studies precluded formal assessment of publication bias. Furthermore, variability in study populations, biomarker definitions, and outcome measures limited the feasibility of meta-analysis and complicated the interpretations. Finally, a moderate to serious risk of bias in most studies together with small sample sizes, inadequate follow-up, and insufficient control for confounders reduced the confidence of the findings.

### 4.1. Implications for Future Research

The findings of this review highlight the need for substantial methodological and conceptual improvements in future research on biomarkers for predicting malignant transformation of premalignant laryngeal lesions. First, study design must evolve towards larger, multi-center collaborative efforts to ensure adequate statistical power and generalizability. Prospective cohort studies with standardized protocols and sufficient follow-up duration are particularly important to generate high-quality evidence. Furthermore, independent validation of promising biomarkers in separate populations should become a priority, alongside the establishment of consensus definitions for malignant transformation.

Methodological rigor also requires enhancement. Future studies must adhere to transparent reporting standards, including detailed documentation of patient flow through the study with reasons for exclusion, extent and patterns of missing data for each biomarker and outcome, comparison of characteristics between complete and incomplete cases, and sensitivity analyses examining the impact of different missing data assumptions on results. Also, future studies require assay standardization. Biomarker measurement methods varied substantially across studies, including differences in antibody clones, staining protocols, scoring systems (e.g., H-score vs. percentage positivity), and cutoff thresholds for positivity. Without validated, standardized assays with established analytical and clinical validity, biomarkers cannot achieve the reproducibility required for clinical implementation.

Establishing uniform protocols requires: (1) consensus guidelines from professional societies defining malignant transformation endpoints; (2) multi-center validation studies using standardized assay protocols; (3) establishment of quality assurance programs for biomarker testing; and (4) determination of clinically meaningful cutoff values through receiver operating characteristic (ROC) curve analysis in adequately powered cohorts. Statistical analyses should employ advanced survival models and consistently report confidence intervals to improve interpretability.

Biomarker development should move beyond single-marker approaches toward integrated strategies. Validation of candidates such as cortactin/FAK, SOX2, NANOG, and immune signatures are critical, but the creation of multi-marker panels may offer superior predictive performance. Combining biomarker data with clinical and histopathological variables in integrated models could further refine risk prediction. Longitudinal studies examining biomarker dynamics over time will provide valuable insights into the transformation processes.

Technological innovation will play a pivotal role in advancing this field. High-throughput multiomic platforms, including genomics, proteomics, and metabolomics, can accelerate the discovery of novel biomarkers. Machine learning and artificial intelligence offer powerful tools for pattern recognition and risk modeling, while liquid biopsy approaches may enable non-invasive monitoring. Additionally, spatial transcriptomics and proteomics can deepen the understanding of the tumor microenvironment and its role in malignant transformation.

### 4.2. Clinical Translation Considerations

For biomarkers to be transmitted successfully from research to clinical practice, several critical aspects must be addressed. First, analytical validity is essential. Biomarker assays must undergo rigorous validation to ensure accuracy and reproducibility, supported by standardized protocols and inter-laboratory quality control measures. Without this foundation, clinical implementation would lack reliability.

Equally important is the demonstration of clinical utility. Biomarkers should not only predict outcomes but also improve patient care when incorporated into decision-making processes. This requires well-designed clinical studies that show tangible benefits, such as enhanced risk stratification or optimized treatment strategies. Economic feasibility is another key consideration. Biomarker-guided management must prove cost-effective compared to current standards of care, which calls for comprehensive health economic evaluations. These analyses should weigh the potential reduction of unnecessary interventions against the costs of biomarker testing. Finally, practical implementation factors must be addressed. The availability of testing platforms, reasonable turnaround times, and seamless integration into existing clinical workflows are essential for real-world adoption. Without these logistical considerations, even the most promising biomarkers may fail to achieve clinical impact.

## 5. Conclusions

The studies included in this review reveal a diverse biomarker landscape for predicting malignant transformation of premalignant laryngeal lesions. Investigated biomarkers span multiple categories, including protein markers such as cortactin, FAK, NANOG, and CSPG4, cell cycle regulators, immune gene signatures and TILs, microRNAs, and signaling pathway molecules. Among these, cortactin and FAK protein expression emerged as the most promising candidates, supported by robust methodology and clear effect estimates, with combined hazard ratios indicating strong predictive value (HR = 5.042, 95% CI: 2.255–11.274). Immune-related biomarkers represent another emerging area of interest, with TIL patterns and immune gene signatures offering novel approaches to risk prediction.

The development of reliable predictive biomarkers, such those which have been identified in this systematic review, could significantly improve the management of patients with premalignant laryngeal lesions enabling pathologists to differ between progressive and non-progressive laryngeal lesions.

However, despite these advances, the overall quality of evidence remains limited. Most studies exhibited a moderate to serious risk of bias, primarily due to inadequate control of confounding factors, small sample sizes, and incomplete reporting. These limitations underscore the need for more rigorous research to validate and translate these findings into clinical practice. In this way, it would be advisable to obtain larger and well-designed cohort studies that could additionally confirm the validity of the predictive biomarkers presented in the current article.

## Figures and Tables

**Table 1:** Summary of main characteristics and results of the included studies.

Study	Study Design	Participants Characteristics	Lesion Characteristics	Biomarker Details	Main Results
Rodrigo et al. 2011 [9]	Single-center retrospective cohort	82 patients; all men; 36–83 years; 5 years FU	Laryngeal dysplasia	FAK and Cortactin expression by IHC	FAK was an independent predictor of laryngeal cancer development (HR = 3.706, 95% CI: 1.735–7.916; *p* = 0.001) and the combination of FAK and cortactin showed superior predictive value (HR = 5.042, 95% CI: 2.255–11.274; *p* < 0.001).
Bergshoeff et al. 2014 [10]	Single-center retrospective cohort	69 patients (57 male, 12 female)	Laryngeal dysplasia	Detection of chromosome 1 and 7 alterations (FISH)	Chromosome instability is associated with malignant progression of laryngeal premalignancies
Rodrigo et al. 2017 [11]	Multicenter retrospective cohorts (exploration and validation)	82 patients (all men; 36–83 years) and 86 patients (79 male, 7 female, 30–87 years); 5 years FU	Laryngeal dysplasia	NANOG expression by IHC	Strong cytoplasmic NANOG protein expression (score 2) significantly correlated with higher cancer development in both cohorts (HR = 1.826; 95%CI, 1.222–2.728; *p* = 0.003)
Villaronga et al. 2018 [12]	Multicenter retrospective cohort	109 patients; 100 male, 9 female; 30–87 years; 5 years FU	Laryngeal dysplasia	FAK and Cortactin expression by IHC	Validation study of Rodrigo et al. 2011 [9]. FAK and/or cortactin expression independently associated with higher risk of malignant progression (HR = 13.91; 95% CI, 4.82–40.15; *p* < 0.001).
Manterola et al. 2018 [13]	Case–control study	66 cases; 59 male, 7 female; 36–89 years.	Laryngeal dysplasia: 42 non-progressing and 24 progressing	Cancer-associated mutation hotspots by NGS. Validation by qPCR	Mutations in *PIK3CA* and *FGFR3* were detected in progressing dysplasia but absent in non-progressing cases. In contrast, mutations in *JAK3*, *MET* and *FBXW7* were found in non-progressing cases but not in progressing.
Granda-Díaz et al. 2019 [14]	Single-center retrospective cohort	94 patients; all men; 36–86 years; 5 years FU	Laryngeal dysplasia	SOX2 expression by IHC	SOX2 expression was the only significant independent predictor of laryngeal cancer development (HR = 3.531, 95% CI 1.144 to 10.904; *p* = 0.028).
Tuncturk et al. 2021 [15]	Case–control study	30 laryngeal lesions	Benign (*n* = 10), dysplasia (*n* = 10), malignant (*n* = 10)	miRNAs expression by RT-qPCR	Hs_miR-183_5p, Hs_miR-155_5p, and Hs_miR-106b_3p identified as transformation markers, whereas Hs_miR-21_5p, Hs_miR-218_3p, and Hs_miR-210_3p might be biomarkers prone to malignancy
Chu et al. 2023 [16]	Case–control study	46 patients; 35 male, 9 female;	Laryngeal dysplasia: 31 non-progressing and 15 progressing	TILs infiltration and pattern in H&E sections. RNA expression (RNAseq panel OIRRA)	High TILs are significantly correlated with a higher risk of malignant transformation. The non-brisk pattern was significantly associated with an 86% reduced risk of malignant progression (OR = 0.16, 95% CI: 0.03–0.5, *p* = 0.008). TILs showed a highly positive correlation with *CCR6, CD83*, *HLA-DPB1*, *MX1* and *SNAI1*, and they were inversely correlated with *CD48*, *CIITA*, *CXCR4*, *FCER1G*, *IL1B*, *LST1* and *TLR8*
Maffini et al. 2024 [17]	Cross-sectional study	46 patients; 35 male, 9 female;	Laryngeal dysplasia: 31 non-progressing and 15 progressing	RNA expression (RNAseq panel OIRRA)	*CCR4*, *HLA-DQA1*, *HLA-F-AS1, TNFRSF17*, *JCHAIN*, *CD79A*, *CA4*, *CD63*, *SRGN*, *FCER1G* genes overexpressed in progressing dysplasia. *CCL20*, *NOTCH3*, *SNAI2*, *PDCD1LG2*, *STAT4*, *RORC* genes upregulated in non-progressing dysplasia
Montoro-Jiménez et al. 2024 [18]	Single-center retrospective cohort	62 patients; all males; 36–83 years; 5 years FU	Laryngeal dysplasia	*PIK3CA* and *SOX2* amplification by qPCR	*PIK3CA* amplification was the only significant independent predictor of laryngeal cancer development (HR = 2.64, 95% CI 1.09–6.37, *p* = 0.031). Furthermore, combined amplification of both the *PIK3CA* and *SOX2* genes was found the strongest predictor of laryngeal cancer (HR = 2.64, 95% CI 1.09–6.37, *p* = 0.031)
Shi et al. 2025 [19]	Single-center prospective cohort	44 patients; 43 male, 1 female; 5 years FU	Laryngeal dysplasia (*n* = 27); No dysplasia (*n* = 5); Carcinoma in situ (*n* = 12)	CSPG4 expression by IHC	CSPG4 overexpression indicates higher risks and shorter time of postoperative leukoplakia recurrence and tumorigenesis. (HR = 2.243, 95% CI 1.041–4.835, *p* = 0.039)

FAK: Focal Adhesion Kinase; IHC: Immunohistochemistry; FISH: Fluorescent In Situ Hybridization; FU: Follow-up; qPCR: quantitative Polymerase Chain Reaction; RNAseq: RNA sequencing; H&E: Hematoxylin and Eosin; TILs: Tissue Infiltrating Lymphocytes; OIRRA: Oncomine Immune Response Research Assay.

## Data Availability

No new data were created or analyzed in this study.

## Supplementary Materials

The following supporting information can be downloaded at: https://www.mdpi.com/article/10.3390/diagnostics16020236/s1, Table S1: Search strategies. Table S2: Excluded articles after initial screening. Table S3: PRISMA 2020 Main Checklist.

## Abbreviations

The following abbreviations are used in this manuscript:

ELS	European Laryngological Society
WHO	World Health Organization
NCCN	National Comprehensive Cancer Network
HPV	Human papillomavirus
FAK	Focal Adhesion Kinase
CTTN	Cortactin
IHC	Immunohistochemistry
FISH	Fluorescent In Situ Hybridization
FU	Follow-up
qPCR	Quantitative Polymerase Chain Reaction
RNAseq	RNA sequencing
OIRRA	Oncomine Immune Response Research Assay
TIL	Tissue Infiltrating Lymphocytes
