# Supplementary material for: Biomarkers for Predicting Malignant Transformation of Premalignant Lesions of the Larynx: A Systematic Review
Source: Diagnostics (Basel). 2026 Jan 12;16(2):236. doi: 10.3390/diagnostics16020236 (PMC12840134; doi:10.3390/diagnostics16020236)
Supplement: Supplementary file 1 [file diagnostics-16-00236-s001.zip › diagnostics-4050196-supplementary.pdf]

SUPPLEMENTARY MATERIAL

Biomarkers for Predicting Malignant Transformation of Premalignant Lesions of the Larynx: A Systematic Review

Supplementary Table S1: Search strategies

**PubMed Search strategy 1; n = 28**
((“biomarkers”[MeSH Terms] OR “biomarkers”[All Fields] OR “biological markers”[All Fields] OR “molecular markers”[All Fields]) AND (“laryngeal neoplasms”[MeSH Terms] OR “larynx”[All Fields] OR “laryngeal”[All Fields]) AND (“precancerous conditions”[MeSH Terms] OR “premalignant”[All Fields] OR “dysplasia”[All Fields] OR “leukoplakia”[All Fields] OR “erythroplakia”[All Fields]) AND (“neoplasm invasiveness”[MeSH Terms] OR “malignant transformation”[All Fields] OR “cancer progression”[All Fields] OR “transformation”[All Fields]))
**PubMed Search strategy 2; n = 20**
((“p53”[All Fields] OR “p16”[All Fields] OR “Ki-67”[All Fields] OR “EGFR”[All Fields] OR “cyclin”[All Fields] OR “PCNA”[All Fields]) AND (“laryngeal”[All Fields] OR “larynx”[All Fields]) AND (“dysplasia”[All Fields] OR “premalignant”[All Fields] OR “precancerous”[All Fields]) AND (“malignant transformation”[All Fields] OR “progression”[All Fields]))
**Scopus Search strategy; n = 27**
"biomarkers" OR "biomarkers" OR "biological markers" OR "molecular markers" AND "laryngeal neoplasms" OR "larynx" OR "laryngeal" AND "premalignant" OR "dysplasia" OR "leukoplakia" OR "erythroplakia" AND "malignant transformation" OR "cancer progression" OR "transformation"
**Embase Search strategy; n = 30**
('biomarkers'/exp OR 'biomarkers' OR 'biological markers'/exp OR 'biological markers' OR 'molecular markers'/exp OR 'molecular markers') AND ('laryngeal neoplasms'/exp OR 'laryngeal neoplasms' OR 'larynx'/exp OR 'larynx' OR 'laryngeal') AND ('premalignant' OR 'dysplasia'/exp OR 'dysplasia' OR 'leukoplakia'/exp OR 'leukoplakia' OR 'erythroplakia'/exp OR 'erythroplakia') AND ('malignant transformation'/exp OR 'malignant transformation' OR 'cancer progression'/exp OR 'cancer progression' OR 'transformation'/exp OR 'transformation') AND [2011-2025]/py
**Google Scholar Search strategy 1; n = 50**
“biomarkers” "malignant transformation" "premalignant laryngeal lesions" “dysplasia”
**Google Scholar Search strategy 2; n = 9**
"laryngeal dysplasia" “biomarkers” "cancer progression" "predictive markers" “transformation”

Supplementary Table S2: Excluded articles after initial screening

Article	Reason
Chen Y, Li C, Su T, Li D, Shi S. Role of SphK1/S1P/S1PR1 Signaling Pathway in the Progression of Vocal Fold Leukoplakia of Patients With Laryngeal Reflux. J Voice. 2024 :S0892-1997(24)00379-5. doi: 10.1016/j.jvoice.2024.10.029.	Wrong study design
Filho FSA, Santiago LH, Fernandes ACN, Korn GP, Pontes PAL, Camponês do Brasil OO. Preliminary Correlation of the Immunoexpression of Cathepsin B and E-Cadherin Proteins in Vocal Fold Leukoplakia. J Voice. 2024 ;38:760-767. doi: 10.1016/j.jvoice.2021.08.005.	Insufficient follow-up
Liu Y-Y, Zhuang P-Y. Combined Transcriptomic and Proteomic Forecast Analyses for Potential Biomarkers of Smoking-Induced Benign and Malignant Transformation of Vocal Fold Lesions. World Journal of Otorhinolaryngology - Head and Neck Surgery 2025. http://dx.doi.org/10.1002/wjo2.70023	Insufficient follow-up
Baran CA, Agaimy A, Wehrhan F, Weber M, Hille V, Brunner K, Wickenhauser C, Siebolts U, Nkenke E, Kesting M, Ries J. MAGE-A expression in oral and laryngeal leukoplakia predicts malignant transformation. Mod Pathol. 2019 ;32:1068-1081. doi: 10.1038/s41379-019-0253-5.	No separation between oral and laryngeal leukoplakia
Cui W, Xu W, Yang Q, Hu R. Clinicopathological parameters associated with histological background and recurrence after surgical intervention of vocal cord leukoplakia. Medicine (Baltimore). 2017 ;96:e7033. doi: 10.1097/MD.0000000000007033.	No biomarker analysis
Álvarez-Marcos C, López F, Alonso-Guervós M, Domínguez F, Suárez C, Hermsen MA, Llorente JL. Genetic and protein markers related to laryngeal epithelial precursor lesions and their neoplastic progression. Acta Otolaryngol. 2013;133:281-90. doi: 10.3109/00016489.2012.732708.	Insufficient follow-up

Supplementary Table S3: PRISMA 2020 Main Checklist.

Topic	No.	Item	Location where item is reported
**TITLE**			
**Title**	1	Identify the report as a systematic review.	Page 1, line 3
**ABSTRACT**			
**Abstract**	2	See the PRISMA 2020 for Abstracts checklist	
**INTRODUCTION**			
**Rationale**	3	Describe the rationale for the review in the context of existing knowledge.	Section 1, Lines 91-97 (pages 2-3)
**Objectives**	4	Provide an explicit statement of the objective(s) or question(s) the review addresses.	Section 1, Lines 99-105 (page 3)
**METHODS**			
**Eligibility criteria**	5	Specify the inclusion and exclusion criteria for the review and how studies were grouped for the syntheses.	Section 2.1, lines 111-132
**Information sources**	6	Specify all databases, registers, websites, organisations, reference lists and other sources searched or consulted to identify studies. Specify the date when each source was last searched or consulted.	Section 2.2, lines 135-143
**Search strategy**	7	Present the full search strategies for all databases, registers and websites, including any filters and limits used.	Section 2.2, Supplementary Table 1
**Selection process**	8	Specify the methods used to decide whether a study met the inclusion criteria of the review, including how many reviewers screened each record and each report retrieved, whether they worked independently, and if applicable, details of automation tools used in the process.	Section 2.3, Lines 144-152

**Data collection process**	9	Specify the methods used to collect data from reports, including how many reviewers collected data from each report, whether they worked independently, any processes for obtaining or confirming data from study investigators, and if applicable, details of automation tools used in the process.	Section 2.4, Lines 155-156

Topic	No.	Item	Location where item is reported
**Data items**	10a	List and define all outcomes for which data were sought. Specify whether all results that were compatible with each outcome domain in each study were sought (e.g. for all measures, time points, analyses), and if not, the methods used to decide which results to collect.	Section 2.5, Lines 169-172
	10b	List and define all other variables for which data were sought (e.g. participant and intervention characteristics, funding sources). Describe any assumptions made about any missing or unclear information.	Section 2.4, Lines 157-167
**Study risk of bias assessment**	11	Specify the methods used to assess risk of bias in the included studies, including details of the tool(s) used, how many reviewers assessed each study and whether they worked independently, and if applicable, details of automation tools used in the process.	Section 2.6, Lines 174-176
**Effect measures**	12	Specify for each outcome the effect measure(s) (e.g. risk ratio, mean difference) used in the synthesis or presentation of results.	Section 2.5, Lines 169-172
**Synthesis methods**	13a	Describe the processes used to decide which studies were eligible for each synthesis (e.g. tabulating the study intervention characteristics and comparing against the planned groups for each synthesis (item 5)).	Section 2.7, Lines 184-185
	13b	Describe any methods required to prepare the data for presentation or synthesis, such as handling of missing summary statistics, or data conversions.	Section 2.7, Lines 185-187
	13c	Describe any methods used to tabulate or visually display results of individual studies and syntheses.	Section 2.7, Lines 185-187

	13d	Describe any methods used to synthesize results and provide a rationale for the choice(s). If meta-analysis was performed, describe the model(s), method(s) to identify the presence and extent of statistical heterogeneity, and software package(s) used.	Section 2.7, Lines 187-189
	13e	Describe any methods used to explore possible causes of heterogeneity among study results (e.g. subgroup analysis, meta-regression).	Section 2.7, Lines 187-189
	13f	Describe any sensitivity analyses conducted to assess robustness of the synthesized results.	Section 2.7, Lines 187-189
**Reporting bias assessment**	14	Describe any methods used to assess risk of bias due to missing results in a synthesis (arising from reporting biases).	Section 2.6, Lines 176-182

Topic	No.	Item	Location where item is reported
**Certainty assessment**	15	Describe any methods used to assess certainty (or confidence) in the body of evidence for an outcome.	Section 2.6, Lines 176-182
**RESULTS**			
**Study selection**	16a	Describe the results of the search and selection process, from the number of records identified in the search to the number of studies included in the review, ideally using a flow diagram.	Section 3.1, Lines 193-199 (figure 1)
	16b	Cite studies that might appear to meet the inclusion criteria, but which were excluded, and explain why they were excluded.	Section 3.1, Lines 195-197 (supplementary table 2)
**Study characteristics**	17	Cite each included study and present its characteristics.	Section 3.1, Lines 197-198; Section 3.2, (table 1)
**Risk of bias in studies**	18	Present assessments of risk of bias for each included study.	Section 3.3, Lines 267-277, Figure 2
**Results of individual studies**	19	For all outcomes, present, for each study: (a) summary statistics for each group (where appropriate) and (b) an effect estimate and its precision (e.g. confidence/credible interval), ideally using structured tables or plots.	Section 3.2.2, Table 1
**Results of syntheses**	20a	For each synthesis, briefly summarise the characteristics and risk of bias among contributing studies.	Section 3.3, Lines 267-277, Figure 2

	20b	Present results of all statistical syntheses conducted. If meta-analysis was done, present for each the summary estimate and its precision (e.g. confidence/credible interval) and measures of statistical heterogeneity. If comparing groups, describe the direction of the effect.	Section 3.4, Lines 279-283
	20c	Present results of all investigations of possible causes of heterogeneity among study results.	Section 3.4, Lines 281-283
	20d	Present results of all sensitivity analyses conducted to assess the robustness of the synthesized results.	Not applicable
**Reporting biases**	21	Present assessments of risk of bias due to missing results (arising from reporting biases) for each synthesis assessed.	Section 3.3, Lines 271-277
**Certainty of evidence**	22	Present assessments of certainty (or confidence) in the body of evidence for each outcome assessed.	Section 3.5, Lines 286-289

Topic	No.	Item	Location where item is reported
**DISCUSSION**			
**Discussion**	23a	Provide a general interpretation of the results in the context of other evidence.	Section 4, Lines 299-342
	23b	Discuss any limitations of the evidence included in the review.	Section 4, Lines 344-357
	23c	Discuss any limitations of the review processes used.	Section 4, Lines 357-365
	23d	Discuss implications of the results for practice, policy, and future research.	Section 4.1, Lines 366-406
**OTHER INFORMATION**			
**Registration and protocol**	24a	Provide registration information for the review, including register name and registration number, or state that the review was not registered.	Section 2, Lines 109-110
	24b	Indicate where the review protocol can be accessed, or state that a protocol was not prepared.	Section 2, Line 110
	24c	Describe and explain any amendments to information provided at registration or in the protocol.	Not applicable

**Support**	25	Describe sources of financial or non-financial support for the review, and the role of the funders or sponsors in the review.	Line 455
**Competing interests**	26	Declare any competing interests of review authors.	Line 460
**Availability of data, code and other materials**	27	Report which of the following are publicly available and where they can be found: template data collection forms; data extracted from included studies; data used for all analyses; analytic code; any other materials used in the review.	Line 458

PRIMSA Abstract Checklist

Topic	No.	Item	Reported?
**TITLE**			
**Title**	1	Identify the report as a systematic review.	Yes
**BACKGROUND**			
**Objectives**	2	Provide an explicit statement of the main objective(s) or question(s) the review addresses.	Yes
**METHODS**			
**Eligibility criteria**	3	Specify the inclusion and exclusion criteria for the review.	Yes
**Information sources**	4	Specify the information sources (e.g. databases, registers) used to identify studies and the date when each was last searched.	Yes
**Risk of bias**	5	Specify the methods used to assess risk of bias in the included studies.	Yes
**Synthesis of results**	6	Specify the methods used to present and synthesize results.	Yes
**RESULTS**			
**Included studies**	7	Give the total number of included studies and participants and summarise relevant characteristics of studies.	Yes
**Synthesis of results**	8	Present results for main outcomes, preferably indicating the number of included studies and participants for each. If meta-analysis was done, report the summary estimate and confidence/credible interval. If comparing groups, indicate the direction of the effect (i.e. which group is favoured).	Yes
**DISCUSSION**			
**Limitations of evidence**	9	Provide a brief summary of the limitations of the evidence included in the review (e.g. study risk of bias, inconsistency and imprecision).	Yes
**Interpretation**	10	Provide a general interpretation of the results and important implications.	Yes
**OTHER**			
**Funding**	11	Specify the primary source of funding for the review.	Yes
**Registration**	12	Provide the register name and registration number.	Yes

From: Page MJ, McKenzie JE, Bossuyt PM, Boutron I, Hoffmann TC, Mulrow CD, et al. The PRISMA 2020 statement: an updated guideline for reporting systematic reviews. MetaArXiv. 2020, September 14. DOI: 10.31222/osf.io/v7gm2. For more information, visit: www.prisma-statement.org
